# Unstable hemoglobin Montreal II uncovered in an adult with unexplained hemolysis exacerbated by a presumed viral infection: a case report

**DOI:** 10.1186/s13256-022-03374-y

**Published:** 2022-04-10

**Authors:** Cesare Medri, Adriana Méndez, Angelika Hammerer-Lercher, Alicia Rovó, Anne Angelillo-Scherrer

**Affiliations:** 1 Department of Hematology and Central Hematology Laboratory, Inselspital, Bern University Hospital, University of Bern, 3010 Bern, Switzerland; 2 Institute of Laboratory Medicine, County Hospital Aarau, Tellstrasse 25, 5001 Aarau, Switzerland

**Keywords:** Hemoglobinopathy, Unstable hemoglobin, Hemolysis, Case report

## Abstract

**Background:**

Unstable hemoglobinopathies are rare inherited disorders of hemoglobin causing a reduction of hemoglobin molecule solubility. This results in an unstable hemoglobin tetramer/globin polypeptide, which precipitates within the red blood cell. Affected red blood cells have a reduced lifespan due to oxidative stress and cellular rigidity, and tend to be phagocytized by spleen macrophages more rapidly. Unstable hemoglobin is frequently under- or misdiagnosed, because its clinical presentation varies broadly. Therefore, testing for unstable hemoglobinopathies is indicated in cases of unexplained hemolytic anemia. However, this approach is not systematically followed in clinical practice.

**Case report:**

A 25-year-old Caucasian man with a recent history of a presumed viral upper respiratory infection was referred to the hematology outpatient clinic because of hemolytic anemia. The patient had scleral icterus, moderate splenomegaly, and mild macrocytic anemia with high reticulocyte count. Unconjugated bilirubin and lactate dehydrogenase were elevated. Haptoglobin was undetectable. Direct antiglobulin test was negative. Blood smear examination revealed anisopoikilocytosis, polychromasia, bite cells, and basophilic stippling, but no Heinz bodies. High-performance liquid chromatography and capillary electrophoresis showed slightly increased hemoglobin A2, normal fetal hemoglobin, and a variant hemoglobin. Deoxyribonucleic Acid sequencing revealed the heterozygous mutation c430delC in the beta-globin gene hallmark of hemoglobin Montreal II and the heterozygous mutation c287C>T in the alpha-globin gene corresponding to hemoglobin G-Georgia, indicative of the not yet described combination of double-heterozygous hemoglobin Montreal II and hemoglobin G-Georgia variants. Hemoglobinopathy Montreal II was here not associated with β-thalassemia syndrome, and carriers did not show ineffective erythropoiesis. In addition to the case report, we provide information about the largest pedigree with hemoglobinopathy Montreal II identified to date.

**Conclusion:**

We emphasize that a transitory acute condition may uncover an underlying inherited red blood cell disorder. In this regard, awareness should be raised among hematologists caring for adult patients that unstable hemoglobinopathies should be considered in the differential diagnosis of unexplained hemolytic anemias.

## Introduction

Unstable hemoglobinopathies are rare inherited disorders of hemoglobin (Hb) [1]. More than 200 unstable Hb variants have been reported [2]. They cause a reduction of Hb molecule solubility, resulting in an unstable Hb tetramer/globin polypeptide, which precipitates within the red blood cell (RBC). Affected RBCs have a reduced lifespan due to oxidative stress and cellular rigidity, and tend to be phagocytized by spleen macrophages more rapidly [3].

Unstable Hb is frequently under- or misdiagnosed, because its clinical presentation varies broadly. Therefore, testing for unstable hemoglobinopathies is indicated in cases of unexplained hemolytic anemia.

**Fig. 1:**
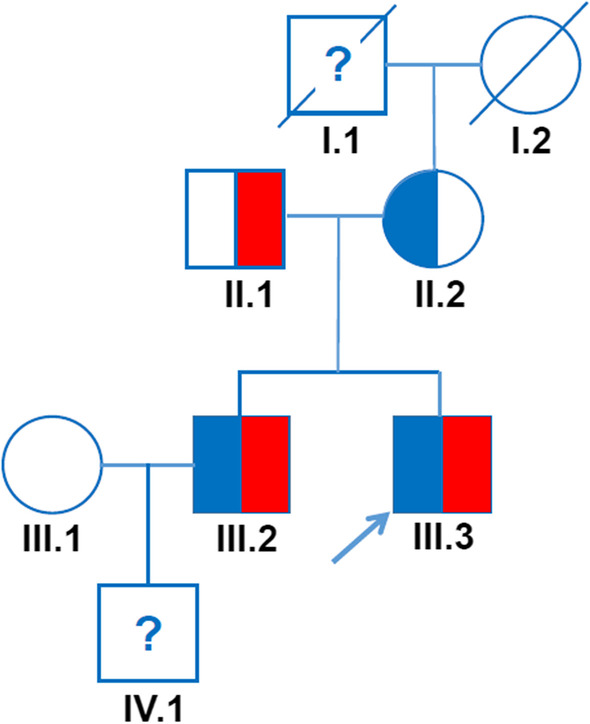
Pedigree of the largest family affected by the unstable hemoglobin Montreal II and by the G-Georgia Hb variant. Unstable hemoglobin Montreal II variant is shown in blue and hemoglobin G-Georgia variant in red. The arrow indicates the index patient

Here, we report a case of unstable hemoglobinopathy in a young adult with unexplained hemolytic anemia, which manifested first during a presumed viral infection, and show his pedigree (Fig. 1). We display the diagnostic steps highlighting the importance of blood smear examination in patients with negative direct anti-globulin (DAT) hemolytic anemia.

## Case presentation

**Fig. 2:**
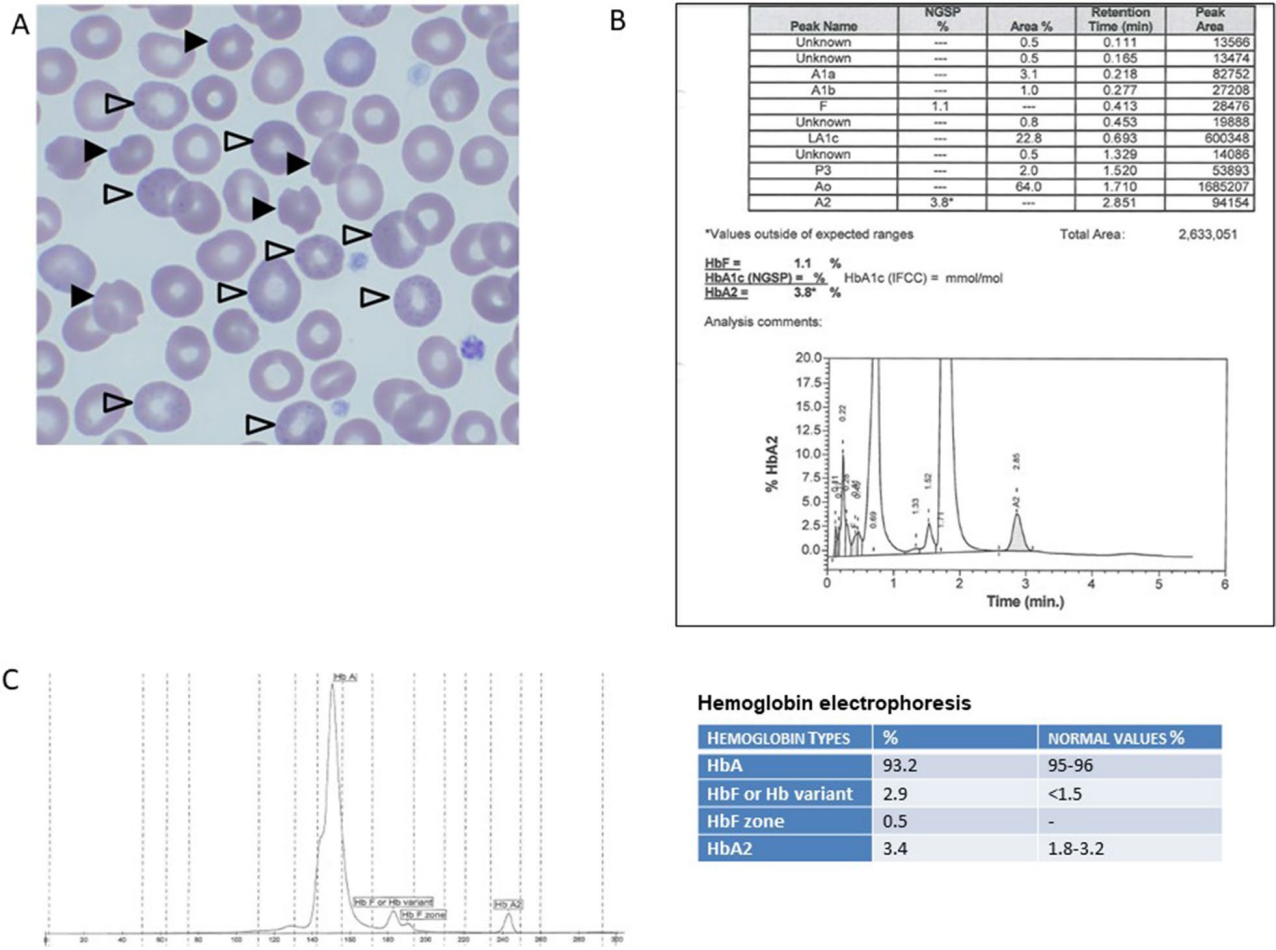
Diagnostic process. **A** Blood smear of the index patient showing bite cells (black arrow head) and basophilic stippling (blank arrow head). **B** High-performance liquid chromatography (HPLC) of the index patient showing the values for HbA2, HbF, and variant hemoglobin (red arrow), with the same retention time as for HbA1c (0.693 minutes) in HPLC. **C** Capillary electrophoresis of the index patient showing two peaks for HbF and an abnormal peak for HbA (adult hemoglobin). These findings are consistent with a high suspicion of a variant hemoglobin

The index case, a 25-year-old Caucasian man (pedigree III.3, Fig. 1) who consulted the otolaryngology outpatient clinic for a presumed viral upper respiratory tract infection, was referred 2 days later to the hematology outpatient clinic because of the suspicion of an hemolytic anemia with an hemoglobin level of 129 g/L. Other than atopic rhinitis, the patient had an uneventful medical history. He started xylometazoline nose drops and saline nasal irrigation 2 days before the hematology consultation in the context of the presumed viral upper respiratory tract infection. In addition, his medication comprised mometasone nasal spray for the atopic rhinitis. The patient had no oral medication, did not smoke, and consumed alcohol occasionally and only moderately. He is a student, living with his parents in Switzerland. Clinical and laboratory characteristics at the time of the hematology consultation are reported in Table 1. Notably, the patient had scleral icterus and moderate splenomegaly. The rest of the physical examination was normal. Laboratory analyses revealed mild macrocytic anemia with high reticulocyte count. Unconjugated bilirubin and lactate dehydrogenase (LDH) were elevated. Haptoglobin was undetectable. DAT test was negative. Blood smear examination revealed anisopoikilocytosis, polychromasia, bite cells, and basophilic stippling (Fig. 2A). However, no Heinz bodies were detected. Although basophilic stippling is not a feature of glucose-6-phosphate dehydrogenase deficiency (G6PD), this diagnostic possibility was investigated and excluded [4], leading us to suspect an unstable hemoglobinopathy. Hb analysis was initiated by cellulose acetate electrophoresis (Helena) and high-performance liquid chromatography (HPLC) (VARIANT II, Bio-Rad Laboratories, Hercules), and confirmed by automated capillary electrophoresis (CapillaryS2 Flex Piercing, Sebia). Sanger sequencing (Applied Biosystems ABI 3500, Thermo Fisher Scientific) was performed using exon-specific primers. HPLC and capillary electrophoresis showed slightly increased HbA2, normal fetal Hb (HbF), and a variant hemoglobin (Fig. 2B, C). DNA sequencing revealed a heterozygous mutation c430delC in the beta-globin gene hallmark of Hb Montreal II [5] and a heterozygous mutation c287C>T in the alpha-globin gene corresponding to Hb G-Georgia [6–11], indicative of the not yet described combination of double-heterozygous Hb Montreal II and Hb G-Georgia variants. Owing to the likelihood that drugs with oxidative effects may aggravate hemolysis in our patient, we recommended him to avoid drugs contraindicated in G6PD deficiency (www.g6pd.org).

**Table 1:** Laboratory and clinical characteristics of the first reported case of Hb Montreal II variant and the largest family affected by the Hb Montreal II and the G-Georgia Hb variants

Characteristics	Normal range (females)	Mother (II.1)	Normal range (males)	Initial case Montreal II	Brother (III.2)	Index patient (III.3)	Father (II.2)	Normal range for age	Nephew (IV.1)
Hb variant	No	Montreal II	No	Montreal II	Montreal II/ G-Georgia	Montreal II/ G-Georgia	G-Georgia	No	NA
Splenomegaly	No	Yes	No	Yes	Yes	Yes	Na	No	NA
Splenectomy	No	No	No	No	Yes	No	No	No	No
RBC (T/L)	3.9–5.0	4.11	4.2–5.7	5.04	3.28	4.66	4.52	4.5–5.3	3.97
Hb (g/L)	121–154	129	135–168	148	89	132	13.9	130–170	104
MCV (fL)	80–98	105	80–98	90.7	87	102	88	71–83	85.6
MCH (pg)	27–33	32	27–33	29.3	27	30	31	23.1–28.2	26.2
MCHC (g/L)	320–360	299	320–360	323	312	305	351	320–359	306
RDW (%)	11.5–14.5	14.1	11.5–14.5	14.9	17.1	13	11.8	11.5–13.1	NA
Reticulocytes (%)	0.5–2.0	9.8	0.5–1.6	6.8	10.3	19.8	1.2	0.5–1.6	NA
Heinz bodies (%)	No	NA	No	1	NA	0	NA	Absent	NA
Bite cells	No	NA	No	Rare	NA	Rare	NA	Absent	NA
Basophilic stippling (%)	No	5	No	10	5	7	0	No	5
LDH (U/L)	< 480	728	< 480	297	582	2342	325	< 480	NA
Conjugated bilirubin (µmol/L)	< 5	13	< 5	6	7	6	6	< 5	NA
Total bilirubin (µmol/L)	< 17	48	< 17	49	22	83	2	< 17	NA
Haptoglobin (g/L)	0.3–2.0	< 0.01	0.3–2.0	< 0.06	NA	< 0.01	1.78	0.3–2.0	NA
AST (U/L)	< 50	27	< 50	NA	22	91	NA	< 50	NA
ALT (U/L)	< 50	18	< 50	NA	27	24	NA	< 50	NA
Ferritin (μg/L)	20–250	174	20–250	NA	771	76	NA	20–250	NA
Pyruvate kinase (U/g Hb)	7.0–13.5	Normal	8.7–16.2	Normal	Normal	Normal	Normal	-	NA
G6PD (U/g Hb)	6.75–11.95	Normal	6.75–11.95	Normal	Normal	Normal	Normal	-	NA
HbA (%)	95–96	Yes	95–96	59.4	97.5	64.0	97.5	-	NA
HbA2 (%)	1.8–3.2	No	1.8–3.2	3.3	2.5	3.8	2.5	-	NA
HbF (%)	< 1.5%	Yes	< 1.5%	2.7	< 1	1.1	< 1	-	NA
Abnormal Hb (%)	No	4.11	No	26.1	0	22.8	0	No	NA

*ALT* alanine aminotransferase, *AST* aspartate aminotransferase, *G6PD* glucose-6-phosphate dehydrogenase, *Hb* hemoglobin, *HbF* fetal Hb, *MCV* mean corpuscular volume, *MCH* mean corpuscular hemoglobin, *MCHC* mean corpuscular hemoglobin concentration, *LDH* lactate dehydrogenase, *NA* not available, *RBC* red blood cell, *RDW* red blood cell distribution width.

The patient had a control at the hematology outpatient clinic 3 weeks later showing a hemoglobin level of 135 g/L and a reticulocyte count of 662 G/L. LDH level was 1843 U/L, indicating the persistence of the hemolytic process. No further follow-up was performed in hematology. Six months ago, a cell blood count obtained at the occasion of a consultation at the allergology outpatient clinic showed a hemoglobin level of 140 g/L. We considered that this was probably the baseline hemoglobin level of our patient.

The grandfather (pedigree I.1, Fig. 1), of Italian origin, had anemia without more specification.

The mother (pedigree II.1, Fig. 1), 66 years old, carries heterozygous Montreal II. She has no anemia, but has signs of chronic hemolysis, had gallstones, and recently underwent cholecystectomy for cholecystitis.

The father (pedigree II.2, Fig. 1), 68 years old, carries heterozygous G-Georgia. His Hb level is normal, and he has no hemolysis.

The brother (pedigree III.2, Fig. 1), 33 years old, carries the combination of double-heterozygous Hb Montreal II and Hb G-Georgia variants. He suffers from moderate anemia and chronic hemolysis, and had splenectomy when he was 23 years old. During the same period, he received a kidney transplant for terminal renal failure because of a rapidly progressive glomerulonephritis.

The nephew (pedigree IV.1, Fig. 1), 3 years old, has mild anemia with basophilic stippling. No further investigation was undertaken.

There is no relevant additional family history to report.

## Discussion and conclusion

Here we report a case of unstable hemoglobinopathy in a young adult with unexplained hemolytic anemia, which manifested first during a presumed viral infection, and show the largest pedigree reported to date of hemoglobinopathy Montreal II. Our case highlights the importance of considering unstable hemoglobinopathy as a differential diagnosis in any adult patient presenting with hemolysis. Notably, what distinguishes our case report from the initial case report of Montreal II hemoglobinopathy report [5] is the circumstance of the diagnosis of the index patient: a presumed viral upper respiratory tract infection that aggravated the hemolysis, drawing the attention of the otorhinolaryngologist consulted to the presumed upper respiratory tract infection and motivating the referral of the patient to hematology consultation. In addition, to our knowledge, the index patient and his brother are the first reported carriers of the combination of double-heterozygous Hb Montreal II and Hb G-Georgia variants.

**Fig. 3:**
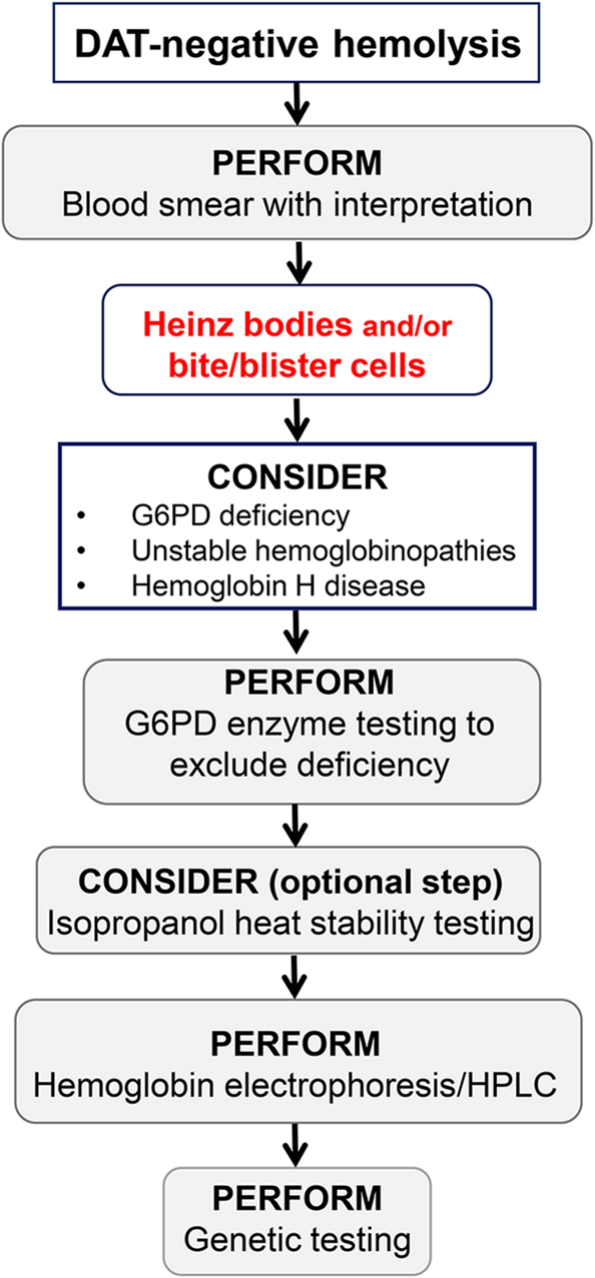
Diagnosis flowsheet. In the context of a direct antiglobulin test (DAT)-negative hemolysis, a blood smear needs to be performed. If Heinz bodies and/or bite cells are present, consider the following differential diagnoses: glucose-6-phosphate dehydrogenase (G6PD) deficiency, unstable hemoglobinopathies, or hemoglobin H disease. Then, perform G6PD enzyme testing. If normal, consider isopropanol heat stability testing. This test is optional; be aware that false positive may occur if hemoglobin F is high or in case of methemoglobinemia [1]. The next step would be hemoglobin electrophoresis/high-performance liquid chromatography (HPLC). Independently of the result, genetic testing is recommended. Indeed, hyperunstable variants may undergo rapid denaturation and degradation, so that the remaining Hb may appear normal. Therefore, normal Hb electrophoresis/HPLC result does not exclude unstable hemoglobinopathy, and genetic testing needs to be conducted [12, 13].

The clinical presentation of unstable Hb depends on the causative mutation. Hemolysis may be chronic or acute, induced by oxidative stress. Chronic hemolysis may be mild to severe, and is usually recognized early in life [12]. Icterus, splenomegaly, and gallstones may belong to the clinical picture. Transfusion might be required during acute hemolytic episodes or chronically in patients with severe hemolysis. Splenectomy is indicated in patients with severe hemolysis and may improve anemia. However, there is a risk of vasculopathy and stroke in the post-splenectomy stage. Guidelines may help with splenectomy decision and choice of splenectomy type [12, 13]. If a hemolytic anemia workup is performed, characteristic findings include decreased Hb and elevated reticulocytes, unconjugated bilirubin and LDH, and undetectable haptoglobin. Facing a patient with DAT-negative hemolysis, the first suspicion of an unstable hemoglobinopathy arises from blood smear examination since it may reveal anisopoikilocytosis, bite and/or blister cells, polychromasia, and basophilic stippling. Heinz bodies may be detected by RBC supravital staining. However, absence of Heinz bodies does not exclude unstable hemoglobinopathy, particularly in nonsplenectomized patients [12]. Indeed, the spleen removes just the RBC membrane parts with Heinz bodies, leaving bite cells. Moreover, Heinz bodies are not specific to unstable hemoglobinopathies and may be observed in other conditions such as G6PD deficiency. Hb stability test may be used as a screening test [1]. In the second diagnostic step, Hb electrophoresis or HPLC may reveal increased HbA2, HbF, and peaks corresponding to abnormal Hb. However, hyperunstable variants may undergo rapid denaturation and degradation, so that the remaining Hb may appear normal. Therefore, normal Hb electrophoresis/HPLC results do not exclude unstable hemoglobinopathy [12, 14]. Finally, diagnostic steps comprise globin gene sequencing, which is the only technique to detect some rare unstable Hb variants [12]. A flowsheet describing the diagnosis process is shown in Fig. 3.

Hb Montreal II is a rare unstable beta Hb variant caused by a single nucleotide deletion that shifts the gene stop codon from codon 147 to codon 157. Presently, only the case of a Canadian patient with compensated chronic hemolysis without thalassemia features has been published (Table 1) [5]. Here, we report a family comprising three heterozygous carriers (pedigrees II.2, III.1, and III.2; Fig. 1) and two possible additional carriers (pedigrees I.1 and IV.1; Fig. 1). Hb Montreal II was here not associated with β-thalassemia syndrome, and carriers did not display ineffective erythropoiesis. Other than pedigree III.2 and the index case at presentation when he had a viral infection, carriers showed compensated chronic hemolysis. Therefore, in absence of additional erythropoietic challenges, heterozygous carriers can compensate for hemolysis by increasing erythropoiesis. The clinical picture of our patient was very similar to that of patients with Hb Tak, Trento, or Saverne [5]. However, Hb Florida, also similar to Hb Montreal II, has been associated with anemia and thalassemia [5].

Hb G-Georgia is an alpha hemoglobin variant caused by a nucleotide mutation (T) at codon 95, causing the substitution of the amino acid proline with a leucine. Heterozygosity may be associated with mild anemia and is asymptomatic [8]. Homozygous individuals display microcytic, hypochromic anemia with mild splenomegaly. Hb G-Georgia was reported in three African-American patients [6–8] and in a Turkish family [10], and has been described in combination with alpha thalassemia [15], HbS heterozygosity [11], and HbC heterozygosity [8].

There are many similarities between our patient and the first reported Montreal II case [5]. Both cases had good health condition and presented with splenomegaly. The Swiss patient was diagnosed shortly after an infection, which could explain the occurrence of severe hemolysis.

There was no clinical and laboratory difference between carriers of heterozygous Montreal II and those of compound heterozygous Montreal II and G-Georgia, confirming that heterozygosity for G-Georgia Hb does not cause relevant RBC changes.

We emphasize the importance of blood smear examination during the diagnosis process of DAT-negative hemolytic anemia. In this regard, awareness should be raised among hematologists caring for adult patients, stressing that inherited RBC disorders remain part of the differential diagnosis in adults and that a transitory acute condition may uncover an inherited RBC disorder. Finally, we want to stress that screening individuals with a family history of unstable Hb is recommended [16].

## Data Availability

The datasets used and/or analyzed during the current study are available from the corresponding author on reasonable request.

## Abbreviations

RBC: Red blood cell
DAT: Direct anti-globulin
LDH: Lactate dehydrogenase
G6PD: Glucose-6-phosphate dehydrogenase deficiency
HPLC: High-performance liquid chromatography
Hb: Hemoglobin
